# Genome-Wide Variant Identification and High-Density Genetic Map Construction Using RADseq for *Platycladus orientalis* (Cupressaceae)

**DOI:** 10.1534/g3.119.400684

**Published:** 2019-09-10

**Authors:** Yuqing Jin, Wei Zhao, Shuai Nie, Si-Si Liu, Yousry A. El-Kassaby, Xiao-Ru Wang, Jian-Feng Mao

**Affiliations:** * Beijing Advanced Innovation Center for Tree Breeding by Molecular Design, National Engineering Laboratory for Tree Breeding, Key Laboratory of Genetics and Breeding in Forest Trees and Ornamental Plants of Ministry of Education, College of Biological Sciences and Technology, Beijing Forestry University, Beijing, 100083, China,; † Department of Ecology and Environmental Science, UPSC, Umeå University, SE-901 87 Umeå, Sweden, and; ‡ Department of Forest and Conservation Sciences, Faculty of Forestry, The University of British Columbia, Vancouver, British Columbia, V6T 1Z4, Canada

**Keywords:** RADseq, linkage map, marker distribution, segregation, genome organization

## Abstract

*Platycladus orientalis* is an ecologically important native conifer in Northern China and exotic species in many parts of the world; however, knowledge about the species’ genetics and genome are very limited. The availability of well-developed battery of genetic markers, with large genome coverage, is a prerequisite for the species genetic dissection of adaptive attributes and efficient selective breeding. Here, we present a genome-wide genotyping method with double-digestion restriction site associated DNA sequencing (ddRAD-seq) that is effective in generating large number of Mendelian markers for genome mapping and other genetic applications. Using 139 megagametophytes collected from a single mother tree, we assembled 397,226 loci, of which 108,683 (27.4%) were polymorphic. After stringent filtering for 1:1 segregation ratio and missing rate of <20%, the remaining 23,926 loci (22% of the polymorphic loci) were ordered into 11 linkage groups (LGs) and distributed across 7,559 unique positions, with a total map length of 1,443 cM and an average spacing of 0.2 cM between adjacent unique positions. The 11 LGs correspond to the species’ 11 haploid genome chromosome number. This genetic map is among few high-density maps available for conifers to date, and represents the first genetic map for *P. orientalis*. The information generated serves as a solid foundation not only for marker-assisted breeding efforts, but also for comparative conifer genomic studies.

Genomic resources, such as whole genome and RNA sequence data as well as DNA markers, provide thorough understanding of species genetic variation, genome organization, and insights of gene expression and regulation. Forest genomic resources are essential in the translational applications of research findings to tree selective breeding, utilization, and conservation. Conifers are the dominant component of forest ecosystems in the Northern hemisphere and due to their economic and ecological importance, substantial genome sequencing resources have been directed to several representative conifer species (*e.g.*, *Picea abies* (Nystedt *et al.* 2013), *Picea glauca* (Birol *et al.* 2013), *Pinus taeda* (Neale *et al.* 2014; Zimin *et al.* 2014), *Pinus lambertiana* (Crepeau *et al.* 2017), and *Pseudotsuga menziesii* (Neale *et al.* 2017)). Additional research efforts are expected to be further dedicated to sequencing more tree species spanning several taxonomic groups.

The Cupressaceae family is one of the largest extant conifer lineages in terrestrial ecosystem with substantial horticultural and forestry values. Cupressaceae has 30 genera with 133 species with world-wide distribution and harboring the world’s largest (*Sequoiadendron giganteum*) and oldest (*Fitzroya cupressoides*) trees. Members of Cupressaceae family are either monoecious or sub-dioecious and rarely dioecious trees or shrubs, and are remarkable for their morphological diversity and broad habitat adaptation (Pittermann *et al.* 2012). Despite their abundance and ecological, economical, and conservation values, our understanding of Cupressaceae genomes is very limited.

Conifer genomes are generally large. The genome size for Pinaceae ranges from 20 – 30 Gb (Pittermann *et al.* 2012; Nystedt *et al.* 2013; Birol *et al.* 2013; Crepeau *et al.* 2017). For non-Pinaceae species, such as Cupressaceae, Taxaceae and Podocarpaceae the estimated genome sizes are in the range of 9.0 – 12.4 Gb (Burleign *et al.* 2012). Even with the advances of next-generation sequencing, the production of full genome sequences and genome assembly for a conifer species is financially and technically prohibitive. An alternative, but not mutually exclusive way to describing the genome of an organism is that of linkage map. Linkage map orders genetic markers and can link phenotypic traits with genomic regions to interpret population and quantitative genetics patterns of variation (Lynch and Walsh 1998). When available, linkage maps can be complementary to genome assembly in providing local fine-scale genomic information (Bernhardsson *et al.* 2019). Construction of a single-tree linkage map in conifers is straightforward owing to the presence of the haploid megagametophyte tissue that is readily available from open-pollinated seed (*i.e.*, no specific crosses are needed). Each megagametophyte sampled from a mother tree represents a distinct haploid meiotic product, allowing simple segregation analysis of maternal alleles.

The construction of a high-density linkage map requires large number of segregating markers. Traditional molecular markers (*e.g.*, amplified fragment length polymorphism (AFLP) and simple sequence repeats (SSRs)) have been useful in studying forest trees genetic diversity and mating systems. However, their number and genomic distribution is somewhat limited considering the large size of conifer gemones. The developed restriction site associated DNA sequencing (RAD-seq) is an efficient and widely used technique to obtain substantial number of markers data even for species lacking prior genomic information (Baird *et al.* 2008), and represents a platform for generating genome-wide polymorphisms for a variety of applications (Davey *et al.* 2011; Parchman *et al.* 2018). Double digest RAD sequencing (ddRAD-seq) is a modified version of RAD-seq, and possesses greater flexibility and robustness in SNP recovery as it uses two restriction enzymes comprising a rare-cutting one and frequently-cutting one and avoids random shearing of the DNA (Peterson *et al.* 2012). Precise and repeatable size selection allows more consistent recovery of shared regions across samples (Peterson *et al.* 2012). ddRAD-seq has been successfully applied to various organisms for the construction of linkage maps, QTL analysis, comparative genomics and genome assembly (Kai *et al.* 2014; Meier *et al.* 2017; Hollenbeck *et al.* 2017; Konar *et al.* 2017).

*Platycladus orientalis* (2n = 22) is a member of the subfamily Cupressoideae, the largest within Cupressaceae. It is native to Korea, Eastern Russia and Northern China, and is globally introduced to many Asian, European, American, and Oceania countries (Li *et al.* 2016). In China, *P. orientalis* grows in a wide range of environmental conditions and is adapted to diverse and extreme climates, and thus considered a keystone species (Wu 1986). Its broad adaptation has made *P. orientalis* the species of choice for major afforestation programs in Northern China (Zhang *et al.* 2016). In addition to the role *P. orientalis* plays in reforestation and environmental protection, the species has substantial economic importance for its extensive use in traditional Chinese medicine and as an ornamental tree. *Platycladus orientalis* has been the subject of limited genetic investigations using SSR markers, including mating system in seed orchard (Huang *et al.* 2018) and genetic diversity in breeding stocks (Jin *et al.* 2016), and impact of future climate change on the species’ distribution (Hu *et al.* 2015). To date, knowledge about *P. orientalis* genome is very limited (Hu *et al.* 2016). According to http://data.kew.org/cvalues/, *P. orientalis* has a C-value of 10.46 pg, which corresponds to a genome size of 10.23 Gb (Gregory *et al.* 2007). For such a large and difficult genome, resources for reduced representation are always good to have. The availability of reliable genetic markers with reasonable genome coverage is considered as a prerequisite for conservation, breeding, and utilization of *P. orientalis*.

The objectives of this study are: 1) to evaluate the applicability of ddRAD-seq for generating a large number of high-quality polymorphic markers in Cupressaceae species, and 2) to construct a high-density genetic map of *P. orientalis* as a first step toward understanding its genome organization. The significances of such effort are three folds: first providing empirical assessment of the genetic property of ddRAD makers (*i.e.*, whether the markers segregate in a Mendelian fashion); second, gaining an overview of *P. orientalis* genome organization and how it compare to other conifer species; and third, generating genomic resources for genetic and breeding applications of *P. orientalis* and related taxa.

## Materials and Methods

### Mapping population and DNA extraction

Open-pollinated seeds and leaves were collected from an elite tree growing in a seed orchard located in Jiaxian, Henan Province, China. Seeds were soaked in water for 24 hr and the haploid megagametophytes were dissected for DNA isolation. Genomic DNA was extracted from 139 megagametophytes and leaves of the mother tree using a plant genomic DNA kit (TIANGEN Biotech Co., Ltd, Beijing) following manufacturer’s protocol. DNA quality and integrity were assessed using 1% agarose gel electrophoresis, and further measured with the ND-2000 spectrophotometer (NanoDrop Technologies, Wilmington, USA).

### ddRAD-seq library preparation and sequencing

To select the suitable restriction enzyme combination and fragment size range of digestion products, we performed an *in silico* analysis using *Pinus taeda* (Zimin *et al.* 2014; Neale *et al.* 2014) as reference. Two restriction enzymes, *Eco*RV and *Sca*I, were selected for ddRAD library construction. *Eco*RV is blocked by overlapping CpG methylation. Given the heavy methylation of repetitive elements in plants, the use of methylation-sensitive restriction enzyme is expected to reduce repetitive content in RAD-seq libraries (Pan *et al.* 2015). The optimal size selection range was set to 414 to 464 bp. This exercise resulted in up to 152,874 fragments in this size range in the pine genome.

A ddRAD-seq library was prepared for the 140 DNA sample (one for the mother tree, 139 from its megagametophytes) following the protocol described by Sun *et al.* (2013) after slight modification. The purpose of including a diploid mother tree sample is to use its genotype as reference to validate the genotype calls of the megagametophytes and improve the accuracy of data filtering. Briefly, 500 ng genomic DNA of each megagametophyte and 2 *μ*g DNA of the diploid tissue were individually digested with *Eco*RV and *Sca*I (New England Biolabs) at 37° for 8 hr followed by a termination step at 65° for 30 min. After digestion, dATP was added to each fragment using a Taq polymerase to produce end-terminal A-overhangs. Next, we performed ligation in each sample with T4 DNA ligase, *Eco*RV-adapters and *Sca*I-adapters at room temperature overnight. The digestion and ligation products of the 140 samples, each with a unique barcode, were pooled in equal volume and purified with E.Z.N.A. Cycle Pure Kit (Omega), followed by a PCR amplification. The PCR products were purified using the E.Z.N.A. Cycle Pure Kit (Omega), and quantified by ND-2000 spectrophotometer. Size selection was conducted on a 2% agarose gel, and DNA fragments in the range of 410 – 470 bp were excised and extracted using a gel extraction kit (Qiagen). Finally, after quality control using an Agilent 2100 Bioanalyzer (Agilent Technologies), the library was pair-end sequenced on an Illumina HiSeq 2500 at a read length of 2 × 125 bp.

### Variant discovery

Sequence reads were first demultiplexed by barcodes with Stacks: process_radtags (Catchen *et al.*, 2013). Sequence read quality was checked using FastQC (Andrews 2010). Low quality reads (average Phred scores <30) and adaptor sequence were removed using Trimmomatic (Bolger *et al.* 2014). We trimmed all reads to 100 bp. All clean reads were then clustered by BLAT (Kent 2002) (-tileSize = 10, stepSize = 5). Sequences with >90% similarity were cataloged into one ddRAD contig/locus. Each ddRAD contig was considered as a bin containing several SNPs. In this study we used the contigs as mapping unit (each regarded as a locus). Due to the haploid nature of megagametophytes, each unique locus should contain one of the possible two alleles of the diploid mother tree genotype. Loci with >2 alleles were identified as repetitive origin and were filtered out. Loci with two segregating alleles in the megagametophyte population were identified as polymorphic. Among the polymorphic loci, we only kept the loci that were in agreement with the mother genotype.

### Genetic map construction and evaluation

We used the Lep-MAP2 (Rastas *et al.* 2015) for marker filtering, grouping and ordering. To be compatible with the input of this program, we set the maternal genotype to 1/2, the paternal genotype to 1/1, and missing genotype to 0/0. The haploid megagametophyte genotype at each locus was converted into a homozygous diploid as input format. The threshold of missing data were set to 20%. Subsequently, markers with significant segregation distortion based on chi-square test (*P* ≤ 0.05) were discarded. Only markers followed the expected 1:1 segregation ratio were used in map construction.

The linkage map was constructed through three steps: “grouping”, “join singles” and “ordering”. Linkage groups (LGs) were identified by a logarithm of the odds (LOD) score threshold using the ‘SeparateChromosomes’ module, with the minimum LOD score set to 5. After an initial grouping of most of the markers into LGs, remaining loci can be added to the identified LGs using the ‘JoinSingles’ module with a LOD score set from 5 to 10. In the ordering step, markers in each LG were ordered repeatedly for 10 times to select the order with the highest likelihood. The initial recombination probability was set at 0.4. We checked for error prone loci (error rate >0.3) inflating the ends of linkage groups, if present, they should be removed, and marker orders re-evaluated. Kosambi function was used to convert recombination value to map distance (Kosambi 1944). The length of each LG was summed to calculate the total length of the map. The generated linkage groups were evaluated with the profile of recombination rate and LOD. The heat maps of linkage relationships (LOD) were produced by CheckMatrix (http://www.atgc.org/XLinkage/MadMapper/). Finally, we used Genetic-Mapper (Bekaert 2016) to plot the linkage map. From the full set of segregating markers, we identified the co-segregating markers (*i.e.*, markers labeled as “duplicates” in the Lep-MAP output file). A set of framework markers were selected by masking the duplicated co-segregating markers. These framework markers were re-ordered to construct the framework map. Synteny between the framework map and full-set markers map was analyzed using shinyCircos (Yu *et al.* 2017).

To evaluate whether the mapped markers were randomly distributed, the linkage groups were divided into 10 cM blocks, and the frequency distribution of marker density per block was assessed. The observed frequencies of the number of markers per block were compared with the expected one from Poisson distribution, *P(x) = e^μ^μ^x^/x*!, where *x* is the number of markers per block and *μ* is the average marker density. Average marker density (*μ*) was used to calculate the expected binomial frequencies for each marker class per block interval for all the linkage groups.

### Data availability

Raw sequences were deposited in the NCBI Sequence Read Archive under project accession number PRJNA510567. File S1 contains the sequence information of the 23,926 loci on the linkage map. File S2 contains the information of linkage map with 23,926 markers, the marker ID, position and the identified co-segregation markers marked as “duplicates”. Supplemental material available at FigShare: https://doi.org/10.25387/g3.9754400.

## Results

### Genome-wide polymorphic marker detection

A total of 366.5 million pair-end reads were obtained from the 140 samples (139 haploid megagametophytes and one diploid tissue). The high-quality base (Q score >30) ratio was 88.4% and guanine-cytosine (GC) content was 35.0%. Among the 139 haploid megagametophytes, the number of reads recovered varied from a minimum of 1,479,008 to a maximum of 4,170,363, with an average of 2.5 million. The diploid sample obtained 18,058,470 reads. After one-to-one alignment, clustering and correction, 281.57 M high quality reads were assigned to 397,226 contigs (loci), of which 280,762 were detected in the mother tree with an average depth of 50×. The number of loci called per individual megagametophyte ranged from 173,010 to 261,952 with an average of 220,321. The average depth of coverage in the megagametophyte population was 11.3×. Among these loci, 108,683, 288,226, and 317 were polymorphic (27.4% of the total reconstructed loci), monomorphic, and repetitive, respectively. Of the 108,683 polymorphic loci, 45,959 loci matched the genotypes of the mother tree.

### High-density linkage map

**Figure 1:**
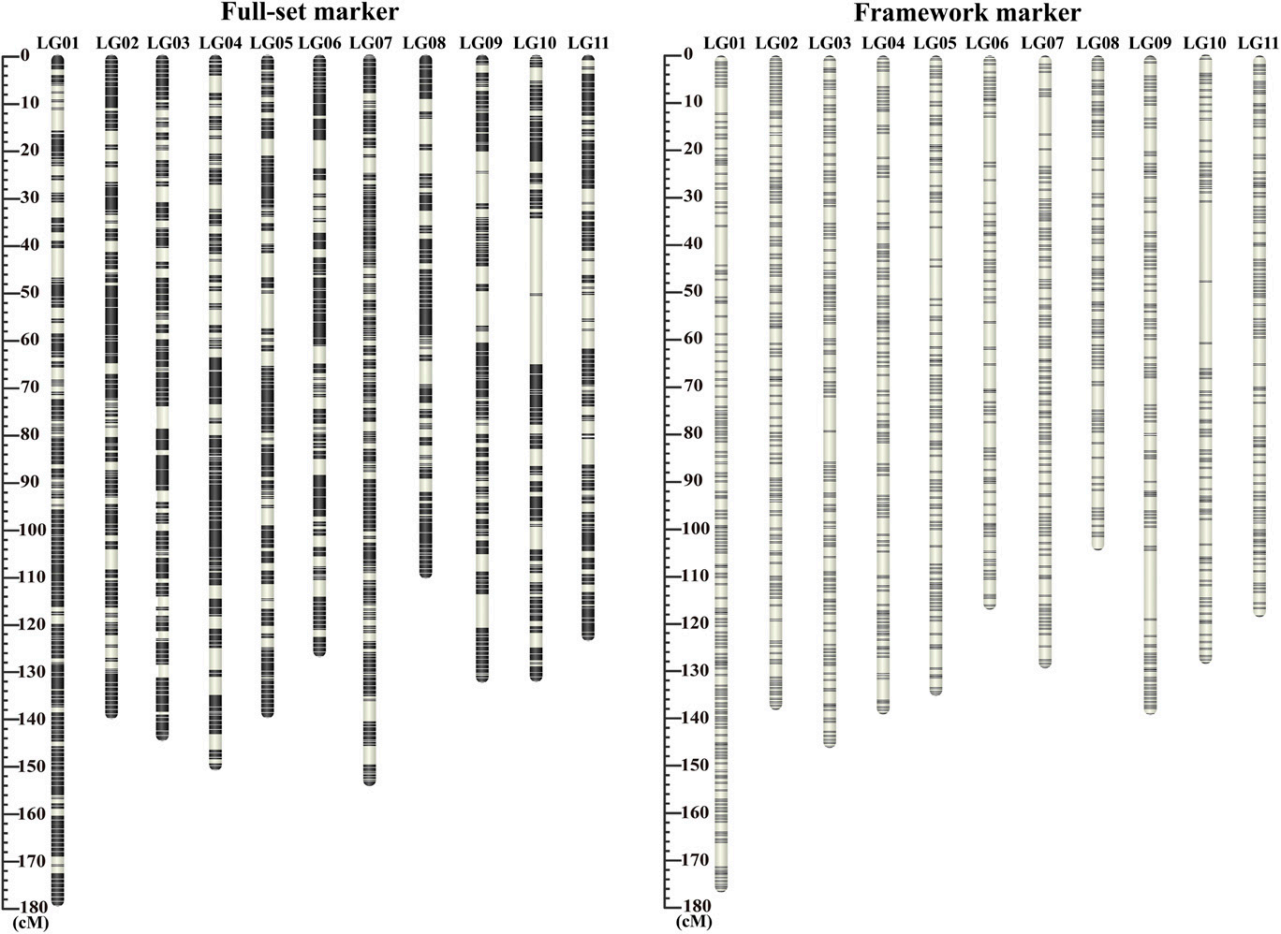
Distribution of full-set and framework markers along the *P. orientalis* 11 linkage groups. The full-set markers map (left) is composed of 23,926 loci with a total length of 1,506 cM. The framework markers map (right) is composed of 7,559 loci with an average spacing of 0.2 cM and a total length of 1,443 cM. In each linkage group, the black horizontal bars show the positions of the markers. The thickness of bar is proportional to the number of co-segregation markers in the same position.

**Figure 2:**
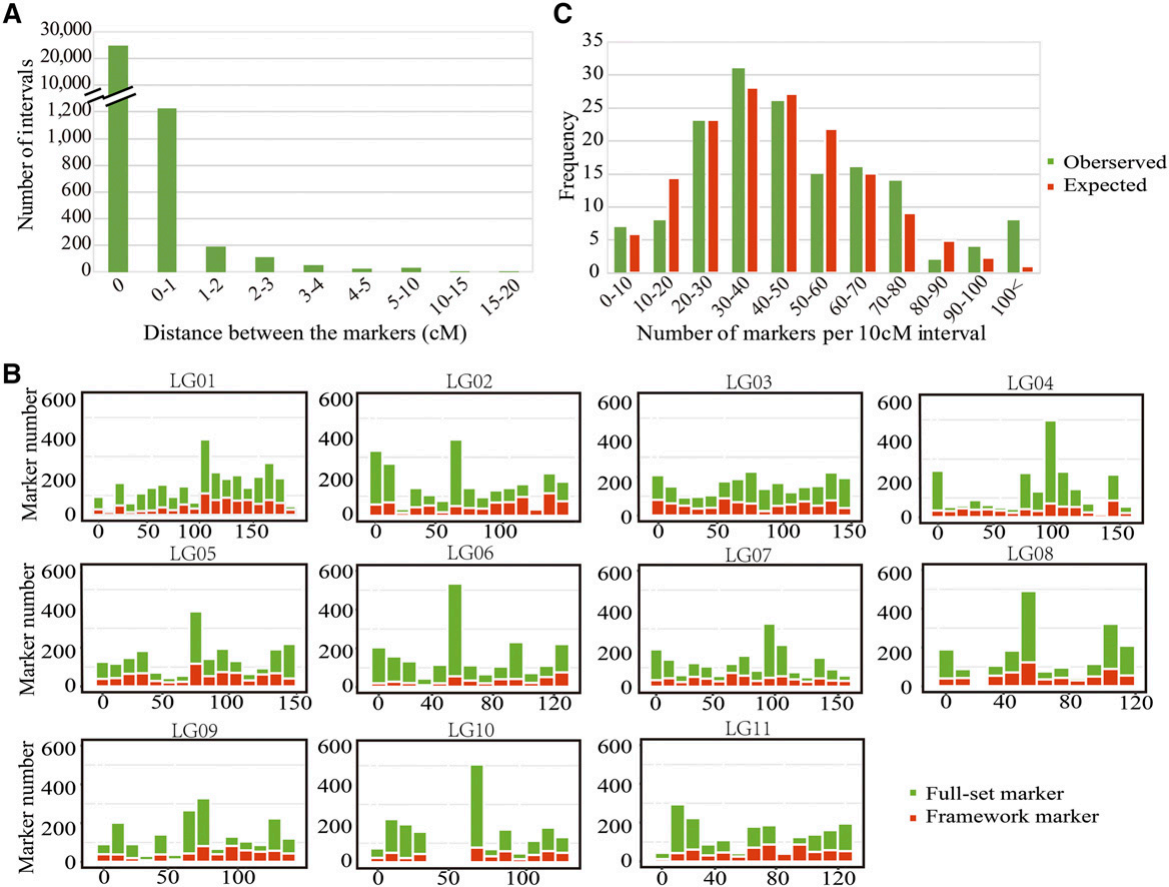
Spatial distribution of marker density along each LG. A) Distribution of the map distance between two adjacent mapped markers. B) distribution of framework and full-set markers along 10 cM sliding windows of each LG. C) Poisson distribution of the observed and expected frequencies of markers distributed at 10 cM interval.

After further filtering away loci with missing rate >20% and segregation distortion (chi-square test, *P ≤* 0.05), 23,926 of the 45,959 polymorphic loci maintained 1:1 segregation ratio in the megagametophyte population and were also found in the mother genotype. These markers were used in the map construction. In the step of grouping, all markers can be successfully classified into 11 linkage groups (Figure 1), corresponding to the *P. orientalis* haploid chromosome number, without executing the ‘JoinSingles’ step. In the ordering step, we did not encounter markers with high error rates at the ends of each LGs. Therefore, the number of mapped markers was the same as the 23,926 1:1 segregating markers. We believe that the reason for this result is the rigorous control of missing rate, error rate and LOD in data filtering, which resulted in high quality markers that can be mapped and ordered without much ambiguity. The final map spanned 1,506.75 cM with the number of markers mapped on each linkage group varied from 1,847 (LG11) to 2,902 (LG01) (Table 1). The length of the linkage groups ranged from 108.35 (LG08) to 176.34 cM (LG01), with an average of 136.98 cM. The distance between two adjacent markers on the linkage groups varied from 0 to 16.48 cM (Figure 2A; Table 1). The maximal gap in each group ranged from 4.9 (LG07) to 16 cM (LG10). Among the 23,915 marker intervals on the 11 LGs, 23,012 intervals were <0.2 cM (96.2%) and 406 intervals were larger than 1 cM (1.7%).

**Table 1:** Summary of the *P. orientalis* linkage map. The full-set of 23,926 segregating markers were ordered into 11 linkage groups. The framework map was constructed using 7,559 markers by masking co-segregating markers

Full-set markers’ linkage map				Framework markers’ linkage map		
Linkage group	Length (cM)	Markers number	Max gap (cM)	Marker number	Length (cM)	Mean interval of markers (cM)
LG01	176.34	2,902	7.2	953	173.19	0.18
LG02	137.47	2,466	5.9	843	135.37	0.16
LG03	142.05	2,394	7.9	862	143.26	0.17
LG04	148.15	2,218	7	637	136.29	0.21
LG05	137.29	2,178	8.2	805	132.47	0.16
LG06	124.62	2,121	9.3	416	114.61	0.28
LG07	151.43	2,033	4.9	650	126.75	0.2
LG08	108.35	1,951	6.3	641	102.29	0.16
LG09	129.97	1,933	9.3	610	136.41	0.22
LG10	129.78	1,883	16.5	466	126.01	0.27
LG11	121.31	1,847	6.6	676	116.13	0.17
**Total**	1,506.75	23,926		7,559	1,442.78	0.2

**Figure 3:**
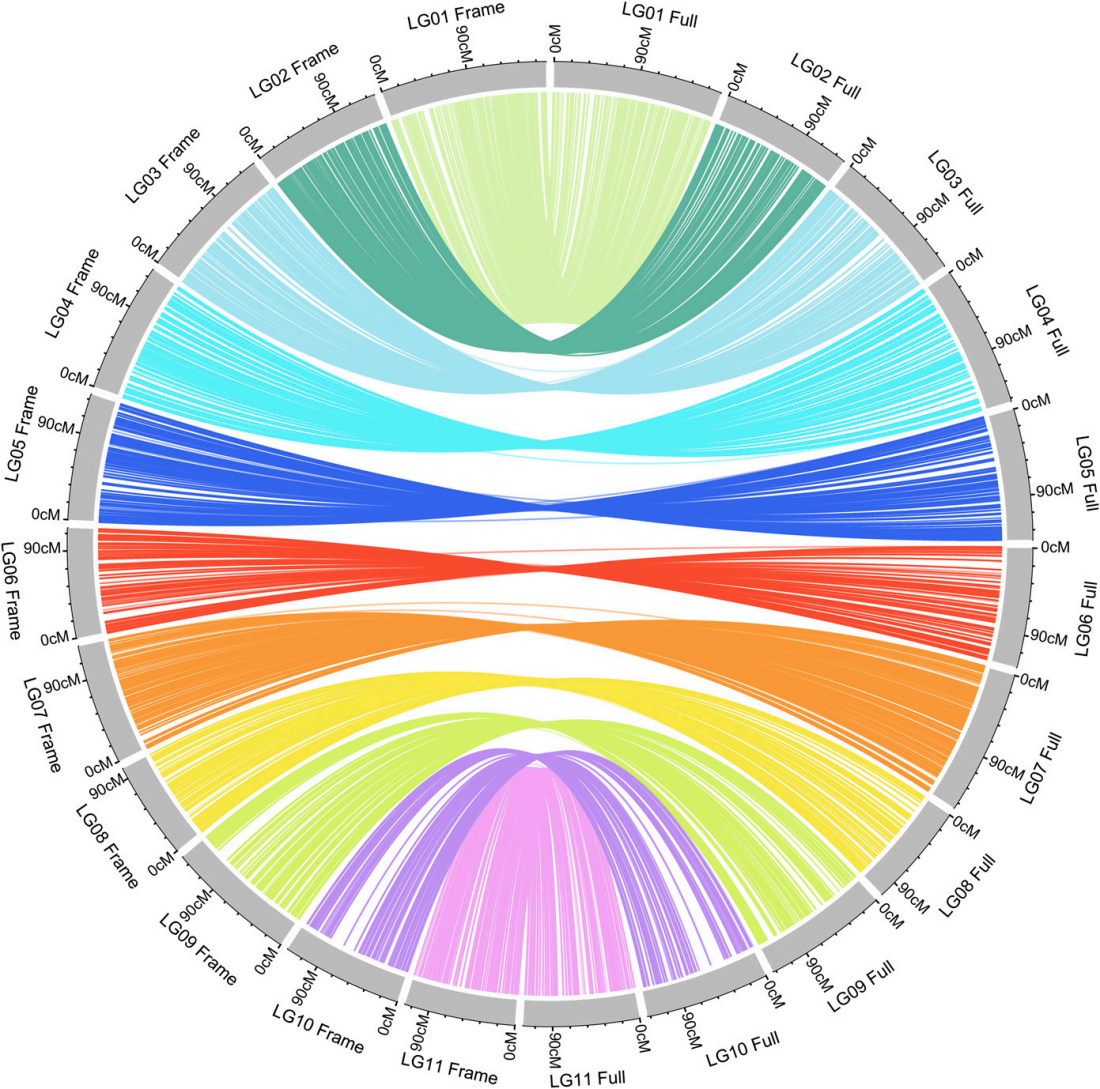
Synteny between the framework marker map and the full-set marker map. Lines connect the same marker on the two maps. Gray blocks on the left represent the 11 linkage groups of the framework map, whereas the 11 blocks on the right represent the full-set marker map.

Among the 23,926 mapped loci, 16,367 were classified as co-segregating loci. Lep-MAP2 identifies “duplicates” based on both map position and consistency in marker order among runs. Markers within a block can be ordered randomly between runs due to small differences in position. Only the consistent marker orders are identified as “duplicates”. We randomly selected one marker from each co-segregating bin and formed a set of 7,559 framework markers. We re-ordered and reconstructed linkage maps using these 7,559 framework markers. No duplicate co-segregation markers were found by the Lep-MAP algorithm in this set of markers. These markers were also grouped into 11 LGs with 416 to 953 loci per group (Figure 1, Table 1). They covered a genome size of 1,442.78 cM, with an average map distance of 0.2 cM between adjacent markers. The sequences of the contigs and detailed information of the markers on each linkage map are available in File S1 and File S2. To compare the synteny between the framework map and the full-set markers’ map, the 7,559 markers in framework map were linked to their positions on the full map. We found highly consistent marker orders between the two maps, as shown in Figure 3. A near-perfect concordance between the two maps suggests good quality marker selection and map ordering.

We examined the distribution of markers in each linkage group and plotted them in 10 cM intervals along the length of each LG. We found a nearly full coverage of markers on each LG, with few regions of low-marker density scattered among the LGs, especially in LG10, which showed a large gap in the region around 40 – 60 cM (Figure 2B). The region with the highest marker density was in the window of 50 – 60 cM on LG06, which contained 525 loci (Figure 2B). The distribution of markers in intervals of 10 cM was in accordance with Poisson distribution, indicating that the markers were randomly distributed in the 11 linkage groups, except for the high end where blocks contained more than 100 markers (Figure 2C).

**Figure 4:**
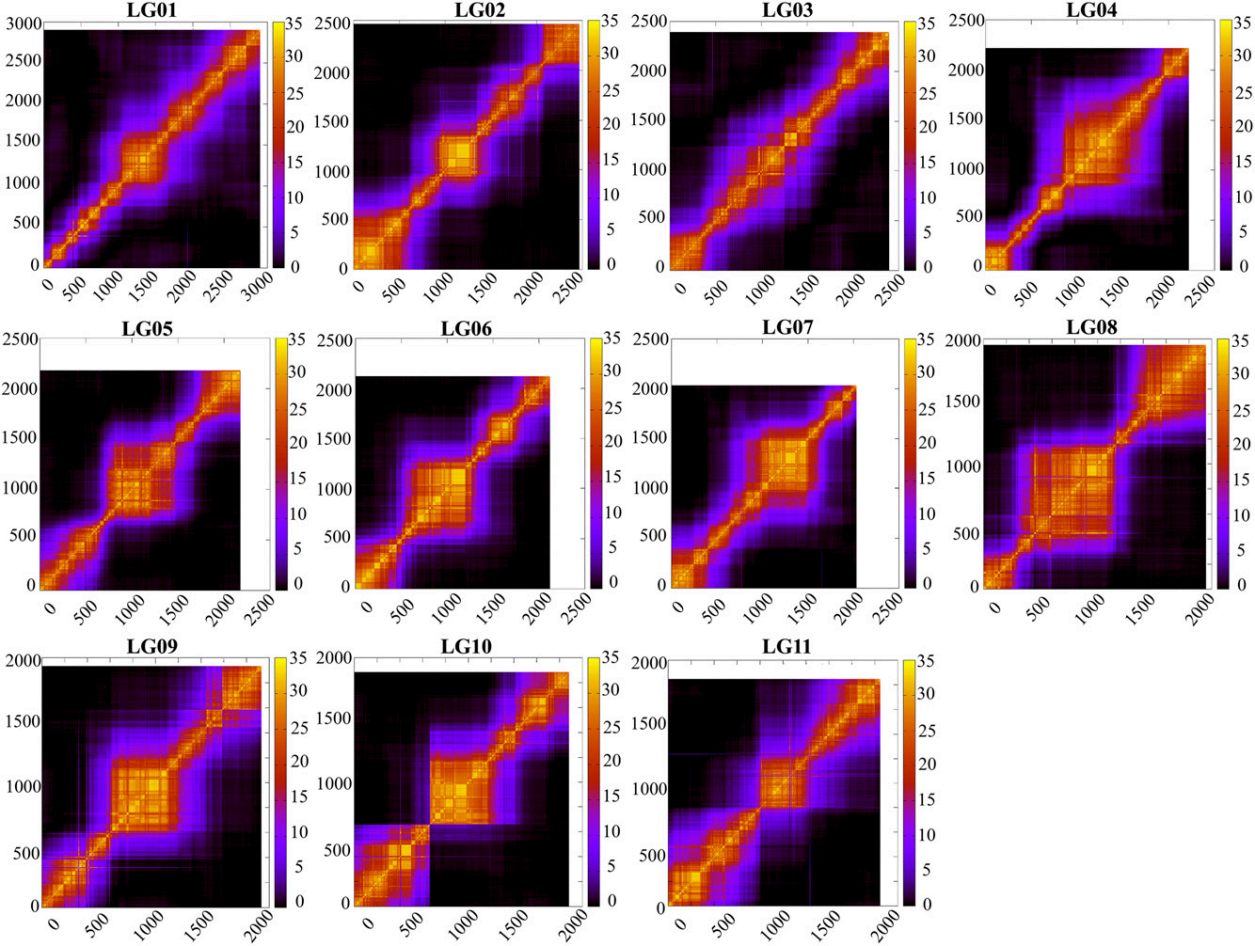
Heat map of the marker linkage relationships in each LG. The plots show the LOD relationship among loci. X- and Y-axes represent the polymorphic ddRAD loci ordered by the linkage map.

The linkage relationship among markers along each LG was visualized as heatmaps of LOD (Figure 4), with higher LOD score indicating tighter linkage. The linkage relationship along each LG differed among LGs with more even spread in *e.g.*, LG01 and LG03, but more clustered in *e.g.*, LG06, LG08 and LG10. This linkage pattern is largely reflective of the marker density distribution on each LG.

## Discussion

We developed a high-density linkage map for *P. orientalis* as a first step toward gaining an insight into the species’ genome organization. The linkage map contains >20K segregating loci covering the entire genome. We demonstrated the efficiency of ddRAD-seq method for genome-wide polymorphism identification in *P. orientalis*, and its utility and potential for other species lacking reference genome for generating large number of markers for genetic studies. In the present study, we assembled 397,226 loci of which 27.4% (108,683) were polymorphic and 72.6% monomorphic in the studied single-tree megagametophyte population. After strict filtering (*e.g.*, in agreement with maternal genotype, 1:1 segregation ratio and discarding markers with >20% missing rate), we obtained 23,926 high-quality loci for linkage map construction.

**Figure 5:**
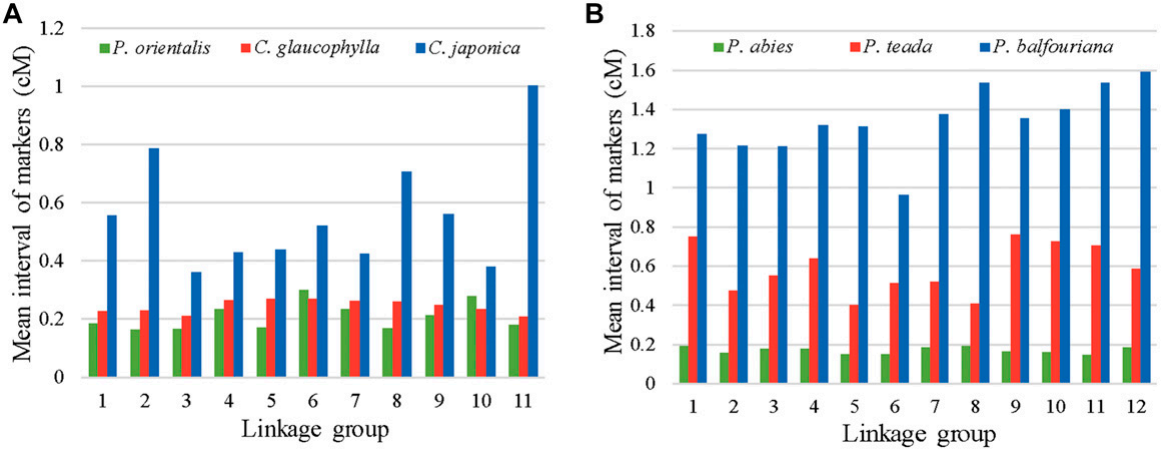
The comparison of average map distances among high-density linkage maps in conifer species. A) The average distance among adjacent markers mapped for *P. orientalis* and two other Cupressaceae species. B) The average distance among adjacent markers mapped for three Pinaceae species.

The linkage map for *P. orientalis* comprised 11 LGs, representing the haploid chromosome number of the species (Sax and Sax 1933), with 23,926 markers covering 1,506 cM. This nearly saturated linkage map is the first genetic map for *P. orientalis*, and to our knowledge is among the densest linkage maps available for conifer species. For example, maps with 21,056 markers spanning 3,556 cM for *Picea abies* (Bernhardsson *et al.* 2019), 20,655 markers spanning 1,192 cM for *Pinus balfouriana* (Friedline *et al.* 2015), 2,841 markers spanning 1,637 cM for *Pinus taeda* (Neves *et al.* 2014), 2,560 markers spanning 1,266 cM for *Cryptomeria japonica* (Moriguchi *et al.* 2016), and 4,284 markers spanning 1,033 cM for *Callitris glaucophylla* (Sakaguchi *et al.* 2015). The average marker interval on the current map of *P. orientalis* is 0.2 cM, which is shorter than most of the maps aforementioned but comparable to the *P. abies* map (Figure 5).

Among the high-density linkage maps available to date, three maps, *P. orientalis*, *C. glaucophylla*, *C. japonica* are representatives of the Cupressaceae family. The *C. glaucophylla* map is constructed using a similar technique, RAD-seq, on megagametophytes (Sakaguchi *et al.* 2015). Comparison of the present *P. orientalis* map to that of *C. glaucophylla* showed that we had more than 5× mapped markers, and 45.79% longer map length (1506 *vs.*. 1033 cM). The large difference in the number of markers recovered between the two studies is likely due to the larger sample size and deeper sequence coverage of *P. orientalis* that permitted capturing more segregating loci. The map for *C. japonica* is based on SNP markers from expressed sequence tag (EST) (Moriguchi *et al.* 2016). A common pattern emerged from the *C. japonica* and the present study is the presence of increased marker density in the center region of each LG, which corresponds to the centromere of each chromosome (Moriguchi *et al.* 2016, Figure 2B), suggesting a large number of co-segregating loci in these regions. The strikingly marker-rich regions in linkage groups may be due to the nonrandom digestion of restriction enzymes and the uneven marker polymorphism and recombination rates along the chromosomes (Petes 2001). Low recombination rate can lead to distorted genetic distances and marker clustering on genetic map (Sakaguchi *et al.* 2015). The high degree of marker clustering around the center of each LGs and the detection of this pattern in *P. orientalis*, *C. glaucophylla*, and *C. japonica* genetic maps suggest the presence of symmetrical karyotypes in the Cupressaceae family. Karyotype analysis of *P*. *orientalis* established its n = 11 chromosomes, of which two are likely satellite chromosomes, where secondary constrictions are located on their long arms (Li and Hsu 1984). On the constructed linkage groups, we detected large gaps in LG10 and LG09 (Figure 2B), which we postulate that it could be secondary constriction regions. Although this assertion cannot be confirmed without a physical map, either way, these regions could act as landmark references for further studies.

High-density genetic maps inevitably generate many co-segregating markers (Iehisa *et al.* 2014; Friedline *et al.* 2015; Sakaguchi *et al.* 2015). The effect of co-segregation markers on linkage maps has received less attention. More mapped markers could capture more recombination events. On the other hand, an excess of co-segregation markers could lead to substantial map expansion due to errors in the data. A study on wheat investigated the effect of segregation markers on map length, which showed that with 10% and 80% co-segregation markers, the map length can be inflated by *ca*. 4% and 12–16%, respectively (N’Diaye *et al.* 2017). In the present study, we generated two maps based on full-set (23,926) and framework markers (7,559), respectively. The map of the full-set markers contained 3× more mapped markers than the framework marker map. The map length, however, is only 4.4% longer than the framework map (1,506 *vs.*. 1,443 cM, Table 1). This result suggests a near saturation of the framework marker map with 7,559 markers. Framework maps have been used in QTL detection (Shao *et al.* 2015; Liu *et al.* 2016), and as a reference to calculate the genetic distances between markers (Ren *et al.* 2012). Linkage maps can further provide valuable information to maker-based population genetic inferences, such as population structure, diversity and relatedness (Sakaguchi *et al.* 2015). In the present study, the map with full-set markers allowed for visualization of uneven marker distribution along each LG and facilitated the detection of centromeres and the genome regions with strong or weak clustering of co-segregation markers. These important features of genome organization cannot be properly detected using framework markers alone.

While the generation of linkage maps from RAD-seq is a complex endeavor due to the inherent stochasticity and error-prone nature of these data, we successfully recovered 108,683 polymorphic loci in the haploid megagametophyte tissues of a single family, and ordered >20K loci into 11 LGs. It should be emphasized that the development of linkage maps based on the relatively low-cost RAD sequencing has unique technical considerations including a substantial amount of missing data as well as a non-uniform distribution of reads over sequenced regions (Beissinger *et al.* 2013). Caution is also needed as low frequency of genotyping error (≤3%) appears as double or multiple recombinants tend to inflate the map length and reduce marker order power (Buetow 1991; Hackett and Broadfoot 2003). Collectively, all elements such as DNA quality, the choice of restriction enzyme, size selection, and sequencing depth can affect the missing rate. In the present study, we performed *in silico* digestion analyses to aid the selection of restriction enzymes, and *Eco*RV and *Sca*I were selected due to their efficiency and the acceptable read depth. The diploid sample of the mother tree which was used as a genotype reference was sequenced at a high coverage depth (50×) to assist genotype calls in megagametophytes. Our rigorous standard for data filtering (eliminating markers with >20% missing data) and discarding of markers with segregation distortion was effective in reducing the inevitable influence of data error and provided reliable markers for constructing our linkage map. Additionally, the use of a single-tree megagametophyte tissue provided large sample size (n = 139) for correctly detecting polymorphisms; however, we were not able to capitalize on the advantage of using megagametophyte tissue from multiple seed donors which undoubtedly allowed observing markers segregation at other loci.

Cupressaceae genetic and genomic studies are urgently needed owing to their ecological, economic, forestry, and horticulture importance. We believe the approach adopted in the present study is a sensible stop-gap approach for providing high quality markers distributed throughout the genome. These markers will be instrumental in assessing the species genetic diversity, development of conservation strategies, association genetics, genomic selection, and ecological genetics studies. The developed genetic map is expected to assist in scaffolds orienting process of the genome assembly of *P. orientalis* and its related Cupressaceae species.
